# Bilateral Image Subtraction and Multivariate Models for the Automated Triaging of Screening Mammograms

**DOI:** 10.1155/2015/231656

**Published:** 2015-07-09

**Authors:** José Celaya-Padilla, Antonio Martinez-Torteya, Juan Rodriguez-Rojas, Jorge Galvan-Tejada, Victor Treviño, José Tamez-Peña

**Affiliations:** ^1^Grupo de Investigación en Bioinformática, Escuela de Medicina, Tecnológico de Monterrey, 64849 Monterrey, NL, Mexico; ^2^Departamento de Investigación e Innovación, Escuela de Medicina, Tecnológico de Monterrey, 64710 Monterrey, NL, Mexico

## Abstract

Mammography is the most common and effective breast cancer screening test. However, the rate of positive findings is very low, making the radiologic interpretation monotonous and biased toward errors. This work presents a computer-aided diagnosis (CADx) method aimed to automatically triage mammogram sets. The method coregisters the left and right mammograms, extracts image features, and classifies the subjects into risk of having malignant calcifications (CS), malignant masses (MS), and healthy subject (HS). In this study, 449 subjects (197 CS, 207 MS, and 45 HS) from a public database were used to train and evaluate the CADx. Percentile-rank (*p*-rank) and *z*-normalizations were used. For the *p*-rank, the CS versus HS model achieved a cross-validation accuracy of 0.797 with an area under the receiver operating characteristic curve (AUC) of 0.882; the MS versus HS model obtained an accuracy of 0.772 and an AUC of 0.842. For the *z*-normalization, the CS versus HS model achieved an accuracy of 0.825 with an AUC of 0.882 and the MS versus HS model obtained an accuracy of 0.698 and an AUC of 0.807. The proposed method has the potential to rank cases with high probability of malignant findings aiding in the prioritization of radiologists work list.

## 1. Introduction

Breast cancer represents nearly one-third of all female cancer cases in the United States [1] and is one of the global leading causes of death among women [2]. Early detection is important for survival, reducing the mortality rate by up to 65% [3–5]. The screening mammography is the preferred early detection method. Based on X-ray imaging, it is effective in visualizing breast tissue abnormalities such as calcifications and masses, architectural distortions associated with the early development of breast cancer [3].

Great efforts have been made to develop computer-aided detection (CADe) and diagnosis (CADx) systems to assist radiologists in interpreting digital mammograms [6]. Commercial CADe systems have been shown to be very sensitive in detecting breast abnormalities [7, 8]. However, these systems tend to have low specificities, reducing the throughput of expert radiologists [9]. Furthermore, the presence of false positives in CADe systems is increasing the number of unnecessary biopsy procedures [9]. On the other hand, CADx systems are helping clinicians in diagnosing complex illnesses (i.e., detection of aneurysms, lung nodules, etc.) in different medical areas [3, 10]. These systems can be used by radiologists as a second opinion and may help in reaching a correct interpretation of abnormal findings [6, 9, 11]. Additionally, triaging mammograms by risk of breast cancer using these systems might reduce the workload of the radiologists.

CADx systems have already been used in breast cancer diagnosis as a means of assessing the risk of a subject to develop or have breast cancer [12, 13]. To do so, features that measure breast abnormalities are extracted from the mammograms and the risk is evaluated. Among these features, high breast density is recognized as risk feature for future development of breast cancer [14, 15]. However, the detection of breast abnormalities can be enhanced by comparing different images of the same subject, either from the same breast at different time points [16] or by asymmetry analyses of the left and right breasts [16, 17]. The latter provides clues about the presence of early signs of tumors (e.g., parenchymal distortion, small asymmetric bright spots, and contrast). Asymmetry analysis consists of two main stages: alignment of the images and detection of asymmetry through a bilateral image subtraction [17].

Current asymmetry detection methods are often based on simple bilateral subtraction techniques [16, 18]. However, breasts are composed of highly heterogeneous and deformable tissue, resulting in an unlikely perfect match between both breasts [19]. Thus, in order to efficiently measure asymmetry, a registration-based alignment must be performed [20]. It is known that the deviations of the normal symmetrical architecture between breast images reduce the rate of false positive detection of masses from digital mammograms [21, 22]. The alignment is improved by using the nipple as a reference point [23]. Coregistration of both breasts using a robust point matching approach and a novel series of features helped to detect abnormal cases with masses [24]. In addition, feature selection was important in a pool of 20 bilateral computed features coupled to a genetic algorithm to select and build an artificial neural network classifier [19]. Therefore, breast asymmetry can be used to assess the risk of breast cancer.

The objective of this work was to provide evidence that features related to asymmetry and derived from bilateral image subtraction maps might be used to automatically classify mammograms sets by their risk of showing breast cancer. This analysis is based on our previous efforts [25], but it is enhanced by the extraction of hundreds of asymmetry-related features and the use of a more robust automated registration algorithm and simplified by the use of a regularization feature selection algorithm. Although it follows an approach similar to the ones used for mass detection [22, 24], our methodology is also evaluated for the detection of calcifications.

## 2. Materials

A total of 1,796 digitalized film mammograms from 449 different subjects were used in this study. From those, 45 were classified as healthy subjects (HS) (mean age of 59.3, SD of 9.8 years), 197 as subjects with malignant calcifications (CS) (mean age of 58, SD of 10.9 years), and 207 as subjects with malignant masses (MS) (mean age of 64.1, SD of 10.1 years). Each subject had the four standard mammograms taken: left and right craniocaudal (CC) and left and right mediolateral oblique (MLO) projections.

In order to avoid problems associated with intrascanner variability [22, 24, 26], all mammograms in this study were obtained from the Howtek dataset of the Digital Database for Screening Mammography public database [27], in which all mammograms were digitalized using a Howtek 960 scanner using a sampling rate of 43.5 micrometers per pixel and a 12-bit depth.

## 3. Methodology

**Figure 1:**
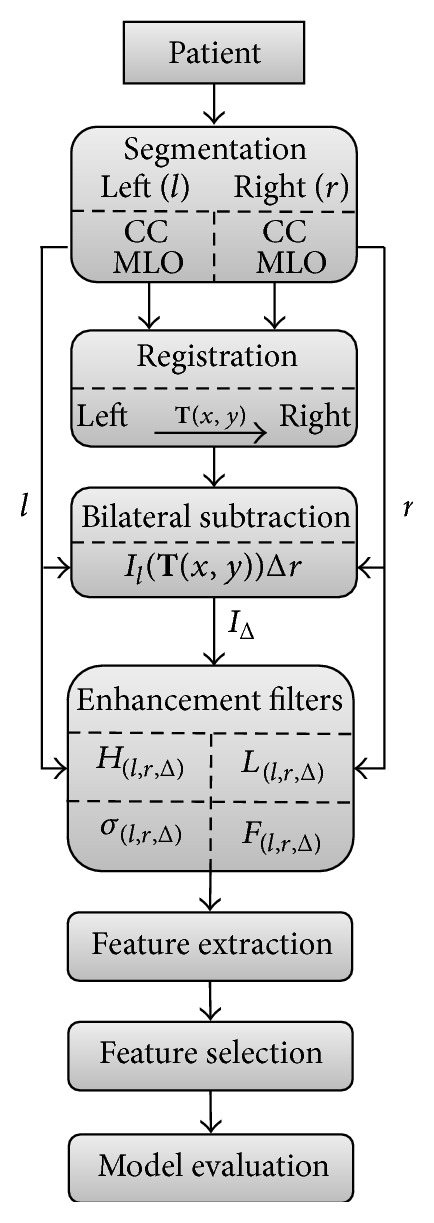
Workflow of the proposed methodology.

Figure 1 shows the seven analysis stages used in this study. Briefly, these stages consist of (1) segmentation of soft tissue, (2) registration of the left images to the right images, (3) bilateral subtraction of the coregistered images, (4) filtering of the images to enhance the signal and texture, (5) feature extraction, (6) multivariate models selection using a train set, and (7) model evaluation on a test set.

### 3.1. Segmentation

**Figure 2:**
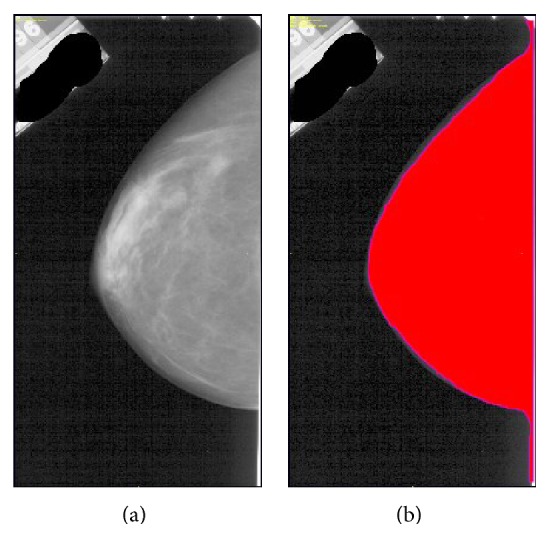
Segmentation of breast tissue: (a) input raw CC view image; (b) segmentation mask of the breast. The red color represents the mask superimposed to the input image.

The automatic segmentation of the breast tissue was based on the estimation of the background noise. An initial segmentation mask was created by estimating the background noise in the image and discarding all pixels below five standard deviations of the noise level. Then, holes were removed by applying closing morphological operations with a 3 × 3 supporting region:

(1) $$\begin{array}{c} S\left(\;A\;\right)=\left(\;A\left(\;x,y\;\right)⊕B\left(\;x,y\;\right)\;\right)⊖B\left(\;x,y\;\right), \end{array}$$

where ⊕ and ⊖ are the grayscale dilation and erosion morphological operations, respectively, *B*(*x*, *y*) is a 3 × 3 structural element, *A*(*x*, *y*) is the image being segmented, and *S*(*A*) is the resulting segmentation of the *A*(*x*, *y*) image. The largest connected region was used as the segmentation mask while all other high intensity regions were removed from the images.  Figure 2 shows an example of the final segmentation using this procedure.

### 3.2. Registration

**Figure 3:**
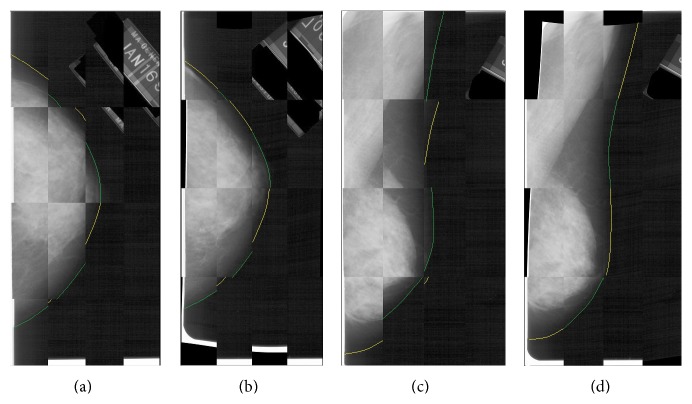
B-Spline image registration example. Checkerboard visualization of (a) unregistered left and right CC views, (b) coregistered left and right CC views, (c) unregistered left and right MLO views, and (d) coregistered left and right MLO views. Yellow (right image) and green (left image) lines were drawn in the edge of the breast for better visualization.

The left breast mammographic images were mirrored and then registered to their corresponding right breast images. The registration used a standard image registration framework with a B-Spline multiresolution transformation optimizing the Mattes mutual information metric [28, 29]. The B-Spline transform **T**(*x*, *y*) deforms an image by modifying a mesh of control points which pinpoints to the moving image to maximize of a similarity measure. Briefly, the images are first registered at the lowest resolution. The B-Spline transformation parameters are then moved into the next resolution and the parameter optimization is run again. The original moving image is lastly deformed using the final parameters of the transform **T**(*x*, *y*). The registration algorithm was implemented using the Insight Toolkit (ITK) libraries [30].  Figure 3 shows an example of this process on an abnormal case.

### 3.3. Image Subtraction

Once the images were coregistered, a pixelwise absolute difference was computed between the left and right images, as follows:

(2) $$\begin{array}{c} I_{\Delta }\left(\;x,y\;\right)=\left|\;I_{r}\left(\;x,y\;\right)-I_{l}\left(\;T\left(\;x,y\;\right)\;\right)\;\right|, \end{array}$$

where *I*_*r*_(*x*, *y*) represents the right image, *I*_*l*_(**T**(*x*, *y*)) represents the left image registered to the right image space, and *I*_Δ_(*x*, *y*) represents the map of absolute differences.

### 3.4. Image Enhancement

To study the appearance of the architectural distortions, two enhancing filters were applied to the images: a morphological high frequency enhancement filter (MoF) designed to enhance fiber-like tissues and a Laplacian of Gaussian filter (LoG) to enhance high frequency patterns inside the breast tissues. Additionally, since the texture between normal and abnormal tissues is different [31], two texture maps were created. The first map computed the local standard deviation (LSD) of the mammographic images and the second map computed the local fractal dimension (LFD). Image processing was implemented in c++ using ITK libraries for image manipulation [30].

#### 3.4.1. Morphological High Frequency Enhancement Filter

The fiber enhancement was done by subtracting the output of the grayscale erosion operation to the original image:

(3) $$\begin{array}{c} H\left(\;x,y\;\right)=I\left(\;x,y\;\right)-I\left(\;x,y\;\right)⊖B\left(\;x,y\;\right), \end{array}$$

where ⊖ represents the grayscale erosion operation, *I*(*x*, *y*) represents the input image, *B*(*x*, *y*) represents a 5 × 5 structural element, and *H*(*x*, *y*) represents the output image. This filter enhances the fine structures by removing the underlying background signal extracted by the grayscale erosion operation [32].

#### 3.4.2. The Laplacian of Gaussian

The image points with high frequency intensity were enhanced using this filter. First convolving the input image using a Gaussian kernel and then applying the Laplacian operator:

(4) $$\begin{array}{c} L\left(\;x,y\;\right)=I\left(\;x,y\;\right)*G_{\sigma }\left(\;x,y\;\right)*h\left(\;x,y\;\right), \end{array}$$

where *G*_*σ*_(*x*, *y*) represents a Gaussian kernel with *σ* standard deviations, *h*(*x*, *y*) represents a 3 × 3 discrete Laplacian operator [32, 33], *I*(*x*, *y*) represents the input image, and *L*(*x*, *y*) represents the output image. Images filtered by the LoG filter will have all its high frequency components enhanced [33]. Therefore, high frequency patterns of the breast tissue will be more prominent after the application of this filter.

#### 3.4.3. Local Standard Deviation

This texture map estimated the per-pixel standard deviation, as follows:

(5) $$\begin{array}{c} \sigma \left(\;x,y\;\right)=\sqrt{\frac{1}{N}\underset{m,n\epsilon R}{\sum }I^{2}\left(n,m\right)-{\left[\frac{1}{N}\underset{m,n\epsilon R}{\sum }I\left(n,m\right)\right]}^{2},} \end{array}$$

where *σ*(*x*, *y*) represents the local standard deviation of the signal within the supporting region *ℜ*(*x*, *y*) of 3 × 3 pixels. This texture map will be bright in areas with large variations in signal patterns, while areas with flat intensities will be black [34].

#### 3.4.4. Local Fractal Dimension

A triangular prism surface area method at three different scales was used to estimate the local fractal dimension at each point of the image. Further details of this methodology can be found elsewhere [35]. The fractal dimension map would have higher values in regions with repeating patterns at the three different resolutions.

### 3.5. Feature Extraction

The enhancement filters and texture maps were applied to the four screening mammography images and to the two bilateral subtraction images, which would result in a set of 15 images for both the CC and the MLO views: *I*_*r*_, *I*_*l*_, *I*_Δ_, *H*_*r*_, *H*_*l*_, *H*_Δ_, *L*_*r*_, *L*_*l*_, *L*_Δ_, *σ*_*r*_, *σ*_*l*_, *σ*_Δ_, *F*_*r*_, *F*_*l*_, and *F*_Δ_, where *I* is the raw image, *H*, *L*, *σ*, and *F* are the enhanced images described in Section 3.4, and *r*, *l*, and Δ stand for the right, left, and bilateral subtraction images, respectively. Features were then extracted from this set of images.

Forty-three features were extracted from each image, resulting in 1,290 image features per subject. Additionally, the average and absolute difference of each left-right pair of measurements was also analyzed, resulting in 860 additional features (symmetric features). Finally, 2,150 features per subject were used.  Table 4 describes the features extracted from each image.

### 3.6. Feature Selection

The first step of the feature selection process consisted of acknowledging for correlations between features. For any pair of features with a Spearman correlation coefficient larger than 0.96, one feature was randomly selected to be kept, and the other was removed from the selection. Two types of normalizations were then applied to this database, resulting in two different datasets. The first dataset was obtained by using a percentile-rank (*p*-rank) normalization and the second one by a *z*-normalization. The empirical distribution of the healthy subjects was considered to perform the *z*-normalization using the rank-based inverse normal transformation [36].

In order to select the most accurate and compact set of features from each dataset, a multivariate search strategy was performed using Least Absolute Shrinkage and Selection Operator (LASSO) [37]. The shrinkage and selection method minimizes the sum of squared errors and penalizes the regression coefficients. The multivariate search was performed using a class balanced data sample of 100 subjects for training and the remaining subjects as a blind test set. Models were calibrated at training using a leave-one-out cross-validation (LOOCV) strategy, training the models at every split using *N* − 1 subjects and evaluating the model using the remaining subject [38]. The final reported performance was obtained by applying the final model gathered in the training stage and evaluating it in the blind test set.

### 3.7. Sensitivity Analysis

The pool of features used in the feature selection procedure was obtained from both the raw and the registered images. However, features gathered from the raw images are side-dependent. Therefore, a sensitivity analysis on the effect of using side-dependent features was performed. Using the final models of HS versus CS, and HS versus MS, left and right side-dependent features were swapped with their contralateral counterparts. Then, we measured the change in model performance on the blind dataset.

## 4. Results

All the 1,796 films were successfully segmented. A total of 9 subject's image sets had to be removed from the experiment due to problems in the registration process. Six were from the malignant mass set, two from the calcification set, and one from the healthy subjects set. All the remaining subjects were included in the subsequent stages of the analysis. The 2,150 extracted features were filtered by the correlation process. This filtering removed 826 due to correlation above 0.96 threshold yielding a total of 1,324 features per subject. These features were analyzed by a univariate statistical analysis of the unadjusted AUC.  Table 1 shows a breakdown of the top 50 features that achieved the highest AUCs.

**Figure 4:**
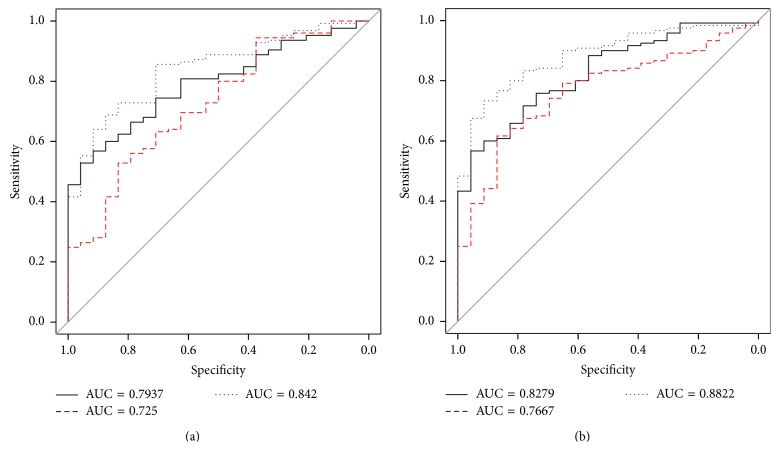
ROC curves of the models generated using the *p*-rank normalized dataset for (a) HS versus MS (AUC = 0.842) and (b) HS versus CS (AUC = 0.882); all performances of the models were measured in blind test set. Red dashed line = different model, black solid line = raw model, and blue dotted line = full model with DIF, SYM, and RAW features.

Table 2 shows the final model for each dataset after features selection. The 21 *p*-ranked features model for the HS versus CS subset achieved a blind accuracy of 0.797 (95% CI 0.692–0.895) and an AUC of 0.882 as shown in Figure 4. The HS versus MS model, containing 12 features, achieved a blind accuracy of 0.772 (95% CI 0.617–0.886) and an AUC of 0.842, as seen in Figure 4.

**Figure 5:**
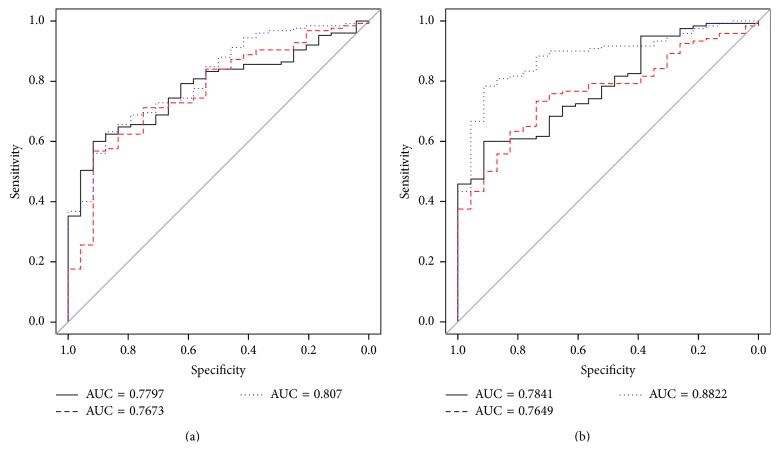
ROC curves of the models generated using the *z*-normalized dataset for (a) HS versus MS (AUC = 0.807) and (b) HS versus CS (AUC = 0.882); all performances of the models were measured in blind test set. Red dashed line = different model, black solid line = raw model, blue dotted line = full model with different, symmetrical, and raw features.

The 12 feature model obtained for the HS versus CS classification, using the *z*-transformation, achieved a blind test accuracy of 0.825 (95% CI 0.727–0.909) and an AUC of 0.882, as shown in Figure 5. The HS versus MS with *z*-transformation generated sixteen features and resulted in a blind test accuracy of 0.698 (95% CI 0.557–0.879) and an AUC of 0.807.  Table 3 shows the complete set of features of each model.

All models included a combination of features extracted from raw, symmetric, and registered images.  Table 3 summarizes the performance achieved by each group of features; Figures 4 and 5 show the difference in performance by each type of features along with the combination of all features (full model).

### 4.1. Cross-Validation and Sensitivity Analysis

**Figure 6:**
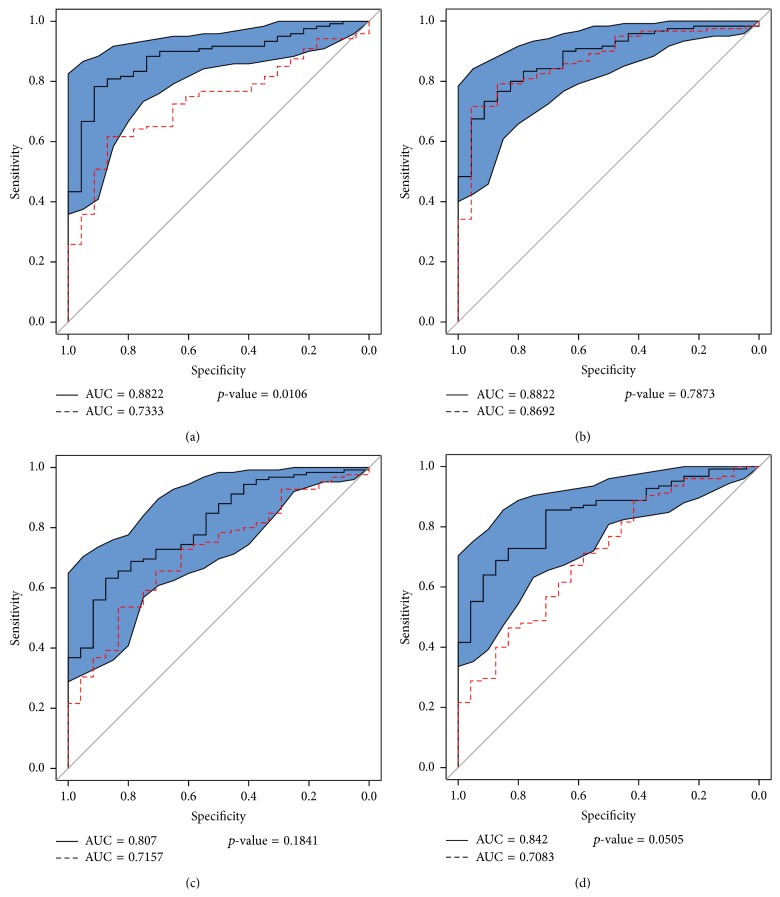
ROC curves for (a) calcifications versus normal model (*z*-normalization), (b) calcifications versus normal model (*p*-rank), (c) mass versus normal model (*z*-normalization), and (d) mass versus normal model (*p*-rank). Solid black line represents the performance of the model, red dashed line represents the model with the swapped raw features, and blue polygon represents the confidence interval of the curve; all performances of the models were measured in blind test set.

Results of sensitivity analysis of the side-dependent features are shown in Figure 6. The blind performance is presented for both the HS versus CS and HS versus MS with raw and swapped features using the *z*-normalized and *p*-rank normalizations.

For the full HS versus CS using *z*-normalization, the AUC decreased from 0.882 to 0.733 by exchanging swapped features. For the HS versus MS, the AUC had a decrease, from 0.807 to 0.760 when exchanging the raw features for the swapped features. AUCs are shown in Figures 6(a) and 6(c), respectively.

When using the *p*-rank normalization, the HS versus CS the AUC decreased from 0.882 to 0.733, and, for the HS versus MS, the AUC dropped from 0.842 to 0.708 when exchanging the raw features to the swapped features (Figures 6(b) and 6(d)), respectively.

The decrease in the AUC when exchanging the raw features for the swapped features was a notable decrease when using the *p*-rank versus the *z*-normalization (15% versus 10%).

## 5. Discussion

### 5.1. Image Processing: Segmentation, Registration, and Digital-Subtraction

The proposed methodology has multiple improvements over other techniques. It is fully automated and does not require manual intervention as previous proposals [21, 22]. Although our approach is similar to others [24], we did not attempt to remove the pectoral muscle from the segmentation mask, since the presence of abnormal axillary lymph in this area is an indicator of occult breast carcinoma [39]. However, from the computational point of view, the feature extraction process may be affected if the region processed is not well focused [40]. The proposed registration process achieved a very good performance and only 2.0% of the subjects had to be discarded due to registration issues. This performance is remarkable in spite of the amount of tissue deformation induced by the mammographic procedure. The spline deformation is an improvement over rigid or affine coregistration methods [26]. The advantage of the deformable registration has been recognized as key element in breast analysis and has been successfully used in longitudinal studies [24]. Regarding digital subtraction, the differences in the X-ray projection and image acquisition and digitizing artifacts may affect the detection of asymmetric patterns. Our results indicate that even in the presence of registration artifacts the digital subtraction added information that was successfully incorporated during the feature selection process.

### 5.2. Feature Extraction

The enhanced images and texture maps enriched the feature set providing a fourfold increase in extracted features per patient, which were also incorporated in the final classification models. Regarding symmetry, the strategy of exploring bilateral symmetry has been explored by other researches where a series of features (signal, texture, breast density, etc.) were computed from each mammogram and the absolute difference between both breasts was obtained to measure breast tissue asymmetry and was used to predict the likelihood of developing cancer [19]. We extended this idea by registering the left and right images using a deformable transformation, which increased the number of features per patient by 25%.

### 5.3. Model Selection and Triaging

The LASSO model selection strategy yields reproducible models of healthy versus malignant mass with a blind test AUC of 0.842 and 0.882 for the detection of malignant calcifications and masses, respectively. Furthermore, the detailed analysis of the ROC curve may give us indications of key triaging points for prioritizing the mammogram reading. Priority triaging may have real practical usage in regions where expert radiological resources are scarce or costly. With the aid of the ROC curve, we may define a low priority reading (10% chance of missing a cancer case: 90% sensitivity). That group represents the 50% and 40% of the screening subjects for malignant calcifications and masses, respectively. On the other side of the spectrum, the high priority reading group may be defined by the 90% specificity cutoff. This 10% of the screening mammograms will have 60% and 50% of the malignant calcifications and masses cases. By assuming that calcification and masses are independent events and by applying the 90% specificity to the 90% sensitivity criteria for triaging, we will have that around 19% (1 − 0.9*∗*0.9) of the subjects will be in a high priority review group, and around 20% (0.4*∗*0.5) of the subjects will be in the low priority review group. The high priority review group will yield around 50% of malignant cases, while the low priority group may have the remaining 10% of the potential malignant cases.

The results of the work cannot yet be generalized and the presented findings are limited to plain film mammograms of a single scanner of the public DDSM dataset. Even having these strong limitations, we believe that the methodology can be replicated with digital mammography or full-field digital mammography and with newer advanced technologies like tomosynthesis. We believe that, in this scenario, our results could be even higher due to the reduction of noise introduced in the mammography scanning and the higher resolution reached.

### 5.4. Sensitivity Analysis

Regarding the side dependency of the raw features as show in Figure 6, the decrease in the AUC when exchanging the raw features for the swapped features when using the *p*-rank versus the *z*-normalization was 15% versus 10%. For the HS versus CS using *p*-rank normalization and HS versus MS using *z*-normalization the differences were not significant (*p*-value = 0.7873 and 0.1841, resp.). However the number of raw-features present in those models was small compared to the length of the model (six of twenty-one and four of sixteen features, resp.); therefore the impact of specific features is limited.

On the other hand HS versus CS using *z*-normalization and HS versus MS using *p*-rank normalization were statistically significant (*p*-value < 0.05) and marginally significant (0.051), respectively, those models had a large proportion of raw features present in the model: six of twelve and five of twelve features, respectively, and the effect of the side-dependency is more noticeable.

Regarding the individual performance of the different feature types, Table 3 shows the individual blind performance along with their combination. In all the cases of the combination of different types of features we observed an increment of the blind AUC; said increments were from a small 2% in HS versus MS using *z*-normalization for the lowest increment to a 9.8% for the HS versus CS using also *z*-normalization. When using the *p*-rank normalization, we observed a similar performance for both HS versus MS and HS versus CS. The HS versus CS achieved the highest AUC; this finding is consistent with the literature [7, 8], since the masses are harder to find [14].

## 6. Conclusion

This proof-of-concept study was able to show that healthy subjects, subjects with calcifications, and subjects with masses can accurately be classified through models generated via accurate mammography registration and a feature selection methodology. The methodology demonstrated that the image subtraction of registered images generates information that improves the identification of subjects with lesions such as malignant masses and calcification. The achieved performance of the system has the potential to be used to queue cases with high chance of malignant findings or may have the practical use of triaging mammograms in developing countries where there is a deficiency of expert readers.

Future work will focus on validating this approach in public databases like the Breast Cancer Digital Repository [41] and exploring the practical implications of translating this methodology to the clinical world.

## Figures and Tables

**Table 1:** Summary of the top 50 univariate features.

Type	*z*-normalization		*p*-rank	
	Healthy vs Calcifications	Healthy vs Masses	Healthy vs Calcifications	Healthy vs Masses
Raw features	25	25	16	24
Symmetric	17	17	20	17
Registered	8	8	14	9

Number of features by type.

**Table 2:** Features of the proposed models.

#	*z*-normalization						*p*-rank normalization					
	Healthy vs Calcifications			Healthy vs Masses			Healthy vs Calcifications			Healthy vs Masses		
	View	Image	Feature	View	Image	Feature	View	Image	Feature	View	Image	Feature
1	CC	*H*_Δ_	27	CC	*L*_Δ_	40	CC	*I*_Δ_	2	CC	*L*_Δ_	40
2	CC	*F*_Δ_	13	CC	*I*_*r*_	29	CC	*F*_Δ_	9	CC	*F*_Δ_	12
3	CC	*I*_*r*_	29	CC	*L*_*l*_	40	CC	*F*_Δ_	12	CC	*I*_*r*_	29
4	CC	*H*_*l*_	29	CC	*F*_*r*_	6	MLO	*I*_Δ_	42	CC	*H*_*r*_	32
5	CC	*H*_*l*_	6	MLO	*H*_*l*_	11	MLO	*I*_Δ_	13	CC	*L*_*r*_	28
6	MLO	*I*_*l*_	28	CC	*I*_Δavg_	29	MLO	*L*_Δ_	32	MLO	*H*_*r*_	32
7	MLO	*H*_*l*_	11	CC	*I*_Δ*s*_	28	MLO	*L*_Δ_	6	MLO	*F*_*l*_	13
8	MLO	*H*_*l*_	21	CC	*σ*_Δ*s*_	38	CC	*I*_*r*_	2	CC	*F*_Δ*s*_	28
9	CC	*I*_Δ*s*_	28	CC	*F*_Δavg_	12	CC	*I*_*r*_	29	CC	*F*_Δavg_	12
10	CC	*σ*_Δ*s*_	38	MLO	*I*_Δ*s*_	40	CC	*H*_*r*_	14	MLO	*I*_Δavg_	2
11	MLO	*L*_Δavg_	27	MLO	*I*_Δ*s*_	28	CC	*F*_*l*_	11	MLO	*I*_Δ*s*_	5
12	CC	*H*_Δ_	27	MLO	*I*_Δ*s*_	29	MLO	*L*_*r*_	15	MLO	*H*_Δ*s*_	39
13				MLO	*H*_Δ*s*_	31	MLO	*F*_*l*_	16			
14				MLO	*H*_Δ*s*_	7	CC	*I*_Δ*s*_	4			
15				MLO	*L*_Δavg_	39	CC	*H*_Δ*s*_	39			
16				MLO	*L*_Δavg_	27	CC	*H*_Δ*s*_	19			
17							CC	*L*_Δ*s*_	12			
18							CC	*σ*_Δ*s*_	17			
19							CC	*F*_Δavg_	12			
20							MLO	*H*_Δ*s*_	31			
21							MLO	*F*_Δavg_	12			

Features are grouped by dataset, symmetric features are denoted with: *I*_Δavg_ = (*I*_*r*_ + *I*_*l*_)/2, *H*_Δavg_ = (*H*_*r*_ + *H*_*l*_)/2, *L*_Δavg_ = (*L*_*r*_ + *L*_*l*_)/2, *σ*_Δavg_ = (*σ*_*r*_ + *σ*_*l*_)/2, *F*_Δavg_ = (*σ*_*r*_ + *σ*_*l*_)/2, *I*_Δ*s*_ = |*I*_*r*_ − *I*_*l*_|, *H*_Δ*s*_ = |*H*_*r*_ − *H*_*l*_|, *L*_Δ*s*_ = |*H*_*r*_ − *H*_*l*_|, *σ*_Δ*s*_ = |*H*_*r*_ − *H*_*l*_|, *F*_Δ*s*_ = |*H*_*r*_ − *H*_*l*_|.

**Table 3:** Model AUC by feature type.

	*z*-normalization		*p*-rank	
	CALC	MASS	CALC	MASS
RAW-features	0.776	0.780	0.828	0.794
DIF-features	0.765	0.767	0.767	0.725
FULL_(RAW+DIF+SYN)_	0.882	0.807	0.882	0.842

RAW stand for raw features, DIF stand for digital subtracted features, SYN stand for symmetric features. The values shown values were obtained using the blind test set.

**Table 4:** 

Category	Feature		Definition
Shape	1	Area	*A*_*s*_
		Perimeter	*P*_*s*_
	2	Compactness	$C_{s}=\frac{P_{s}^{2}}{A_{s}}$
	3	Elongation	$E_{s}=\frac{\underset{}{\max}\left(L_{x}{,L}_{y}\right)}{\min\left(L_{x}{,L}_{y}\right)}$
	4, 5	Region centroid (*n* = 2)	$\hat{x}=\langle \hat{x}=\frac{m_{10}}{m_{00}},\hat{y}=\frac{m_{01}}{m_{00}}\rangle$
	6, 7, 8	Region scatter (*n* = 3)	${RS}_{S}=\left\{\frac{m_{20}}{m_{00}}-{\hat{x}}^{2},\frac{m_{11}}{m_{00}}-\hat{x}\hat{y},\frac{m_{02}}{m_{00}}-{\hat{y}}^{2}\right\}$

Signal	9	Mean	$\mu_{s}=\overset{\underset{}{\max}\left(I\right)}{\underset{i=0}{\sum }}if\left(i\right)$
	10	Median of the Signal	Median = *v*(0.5)
	11	Energy	$E_{s}=\overset{max(I)}{\underset{i=0}{\sum }}i^{2}f\left(i\right)$
	12	Variance	*σ*_*s*_^2^ = *E*_2_ − *μ* ^2^
	13	Standard deviation	$\sigma_{s}=\sqrt{\sigma_{s}^{2}}$
	14	Dynamic range	${DR}_{s}=\underset{}{\max}\left(I\left(m,n\right)\right)-min\left(I\left(m,n\right)\right)$
	15	*z* mean	${\hat{z}}_{s}=\frac{\mu_{s}}{\sigma_{s}}$
	16	Entropy	$H_{s}=\overset{max(I)}{\underset{i=0}{\sum }}\log\left(f\left(i\right)\right)f\left(i\right)$
	17	Skewness	$\gamma_{s}=\overset{max(I)}{\underset{i=0}{\sum }}\left[{\left(\frac{i-\mu_{s}}{\sigma_{s}}\right)}^{3}f\left(i\right)\right]$
	18	Kurtosis	$\beta_{s}=\overset{max(I)}{\underset{i=0}{\sum }}\left[{\left(\frac{i-\mu_{s}}{\sigma_{s}}\right)}^{4}f\left(i\right)\right]-3$
	19	*z* Range	$zDR=\frac{{DR}_{s}}{\sigma_{s}}$
	20, 21	fraction greater than *z* deviations (*n* = 2)	*p*_*z*_ = {1.0 − *F*(*μ*_*s*_ + *zσ*_*s*_) : *z* ∈ {2,3}}
	22, 23	fraction lower than *z* deviations (*n* = 2)	{*F*(*μ*_*s*_ − *zσ*_*s*_) : *z* ∈ {2,3}}
	24–33	Value at fraction (*n* = 10)	vp=vp:p∈0.0001,0.001,0.01,0.05,0.25,0.75,0.95,0.99,0.999,0.9999
	34	5% Trimmed Mean	$\mu_{s}^{5\%}=\overset{v(0.95)}{\underset{i=v(0.05)}{\sum }}if\left(i\right)$
	35	5% Trimmed Standard deviation	$\sigma_{s}^{5\%}={\left[\overset{v(0.95)}{\underset{i=v(0.05)}{\sum }}{\left(i-\mu_{s}^{5\%}\right)}^{2}f\left(i\right)\right]}^{1/2}$
	36	5% Trimmed *z* Mean	${\hat{z}}_{s}^{5\%}=\frac{\mu_{s}^{5\%}}{\sigma_{s}^{5\%}}$

Morphology	37	Total Signal	Mass = *M*_0,0_
	38, 39	Signal centroid (*n* = 2)	$x_{I}=\langle {\hat{x}}_{I}=\frac{M_{10}}{M_{00}}{,\hat{y}}_{I}=\frac{M_{01}}{M_{00}}\rangle$
	40, 41, 42	Signal scatter (*n* = 3)	${RS}_{I}=\left\{\frac{M_{20}}{M_{00}}-{\hat{x}}_{I}^{2},\frac{M_{11}}{M_{00}}-{\hat{x}}_{I}{\hat{y}}_{I},\frac{M_{02}}{M_{00}}-{\hat{y}}_{I}^{2}\right\}$
	43	Signal Surface	$A_{I}=\Delta_{xy}\underset{\left(m,n)\in S(k\right)}{\sum }{\left({\Delta_{xy}}^{2}+{4*\left(I\left(m,n\right)-\hat{I}\left(m,n\right)\right)}^{2}\right)}^{1/2}$

## Appendix

### Image Features

From the segmented image, we can extract a set of features related to segmentation shape, signal distribution, signal texture, and signal morphology. This section describes the equations required in the computation of the image features.

Let us call the segmentation *s*(*m*, *n*) and *I*(*m*, *n*) the digital image. We will define *S*(*k*) as the set of order points of the digital image belonging to a specific tissue *k*:

(A.1) $$\begin{array}{c} S\left(\;k\;\right)=\left\{\;\left(\;m,n\;\right)\in Z^{2}:s\left(\;m,n\;\right)=k\;\right\}, \end{array}$$

where each digital point (*m*, *n*) is associated with a physical coordinate: **x**(*m*, *n*) = 〈*m*Δ*x* + *x*_0_, *n*Δ*y* + *y*_0_〉 (Δ*x* and Δ*y* are the pixel spacing, and (*x*_0_, *y*_0_) is the position of the lower left corner of the image). The physical coordinate represents the location in millimeters of a specific tissue point in the imaging device. The area of the segmented tissue in square millimeters is

(A.2) $$\begin{array}{c} A_{s}=a\underset{\left(m,n\right)\in S\left(k\right)}{\sum }s\left(\;m,n\;\right), \end{array}$$

where *a* is the unit area of a pixel and it is given by *a* = Δ*x*Δ*y*. The perimeter, *P*_*s*_, of the segmentation is the sum of the line segments lengths that join two neighboring edge pixels:

(A.3) $$\begin{array}{c} P_{s}=\overset{L}{\underset{i=0}{\sum }}D, \end{array}$$

where $D=\left\{\left|x_{i}-x_{j}\right|:\left(x_{i},x_{j}\right)\in {ℵ}^{2},{\overset{-}{x}}_{j}\in N_{i}\right\}$ is the set of distances between neighboring edge points. *ℵ* ^2^ is the set of the *L* unordered pairs of the edge points coordinates. |**x**_*i*_ − **x**_*j*_| is the Euclidian distance between the edge point **x**_*i*_ and its neighboring edge point **x**_*j*_.

The density of intensities are given by the normalized histogram:

(A.4) $$\begin{array}{c} f\left(\;i\;\right)=\frac{a}{A_{s}}\underset{\left(m,n\right)\in S\left(k\right)}{\sum }\left[\;I\left(\;m,n\;\right)=i\;\right]. \end{array}$$

The fraction of points lower than a given intensity is

(A.5) $$\begin{array}{c} p\left(\;I<v\;\right)=F\left(\;v\;\right)=\overset{v}{\underset{i=0}{\sum }}f\left(\;i\;\right). \end{array}$$

Hence the upper value of the intensity that contains a given fraction *p* of pixels is

(A.6) $$\begin{array}{c} v\left(\;p\;\right)=F^{-1}\left(\;p\;\right)=\underset{v}{\arg\;\min}\left(\;\left|\;F\left(v\right)-p\;\right|\;\right). \end{array}$$

The moment generating function of the segmentation shape is

(A.7) $$\begin{array}{c} m_{i,j}=\underset{\left(m,n\right)\in S\left(k\right)}{\sum }{\left|\;m\Delta x+x_{0}\;\right|}^{i}{\left|\;n\Delta y+y_{0}\;\right|}^{j}, \end{array}$$

while the moment generating function of the signal morphology is

(A.8) $$\begin{array}{c} M_{i,j}=\underset{\left(m,n\right)\in S\left(k\right)}{\sum }I\left(\;m,n\;\right){\left|\;m\Delta x+x_{0}\;\right|}^{i}{\left|\;n\Delta y+y_{0}\;\right|}^{j}. \end{array}$$

The lengths of the bounding box of the segmentations are given by

(A.9) Lx=maxm⁡xm,n·1,0−minmxm,n·1,0;∀m,n∈Sk,Ly=maxn⁡xm,n·0,1−minnxm,n·0,1;∀m,n∈Sk.

From the above definitions, Table 4 shows the extracted features for each segmented image.
